# What constitutes good care in the context of depopulating rural Korea? Perspectives of village care managers amid digital transitions

**DOI:** 10.1093/geroni/igag018

**Published:** 2026-02-15

**Authors:** Jane Park, Soong-nang Jang, Jongnam Hwang

**Affiliations:** ASEM Global Ageing Center, Seoul, Korea; Red Cross College of Nursing, Chung-Ang University, Seoul, Korea; Department of Health Administration, Wonkwang University, Iksan, Korea

**Keywords:** Integrated care, Digital care quality, Rural health, Digital technology, Smart care

## Abstract

**Background and Objectives:**

Korea is undergoing one of the fastest demographic transitions among Organisation for Economic Co-operation and Development (OECD) countries, with rural communities experiencing the sharpest consequences of aging and depopulation. In these areas, older adults face multiple disadvantages, including social isolation, limited income and education, and restricted access to health and welfare services. In response, national policies have promoted smart care technologies—sensors, monitoring devices, and AI-based services—as part of community-based integrated care. Although these technologies are expected to reduce service gaps, less attention has been paid to how frontline providers understand what constitutes “good care” in this context.

**Research Design and Methods:**

Our study examined the perspectives of village care managers, who coordinate health, welfare, and smart care resources in rural communities. Sixteen managers involved in a pilot smart integrated care project in Jeongeup, Jeonbuk-do, were interviewed through semi-structured, in-depth interviews.

**Results:**

Our findings suggest that good care cannot be solely achieved through technological monitoring or efficiency. It still requires relational qualities—sincerity, respect, and social connection—combined with professional competence and coordination across fragmented services. Participants highlighted that smart technologies may support safety and access but cannot substitute for human interaction, local collaboration, and skilled guidance in bridging digital divides.

**Discussion and Implications:**

By foregrounding providers’ perspectives, this study adds to ongoing debates about the meaning of good care in digitally integrated rural care settings. Smart technologies can support safety and access, but their value depends on how they are embedded in practices of empathy, respect, professional guidance, and community collaboration.

Innovation and Translational Significance: Our study uniquely explores how village care managers, frontline coordinators of health, welfare, and smart care services, define “good care” in depopulating rural Korea amid rapid digitalization. Using qualitative interviews and Tronto’s ethics of care, our results show that technology alone cannot ensure quality care. Good care requires relational qualities—respect, social connection, professional competence, and collaboration with local human resources—integrated with digital tools. These findings guide policy and practice by showing how rural care models can combine smart technologies with human engagement to reduce service fragmentation, bridge digital divides, and build sustainable, person-centered systems for communities.

## Background and objectives

The global phenomenon of rapid aging presents a significant socioeconomic challenge, and Korea is also undergoing rapid population aging. As of 2025, individuals aged 65 and older comprised 20.3% of Korea’s population, a figure projected to exceed 30.0% by 2036 (Korean Statistical Information Service, 2025). This rapid transition, among the fastest in the Organisation for Economic Co-operation and Development (OECD) countries, makes Korea a critical case for examining the implications of aging. Aging is inevitably accompanied by a decline in health and functional abilities, leading to a substantial increase in the number of older individuals requiring care (Ismail et al., 2021). Compounding this issue is the declining availability of caregivers, driven by shifts in family structure and an overall increase in the number of older adults requiring care (Hwang & Lee, 2024). These challenges are especially acute in rural regions, where outmigration of younger residents has accelerated depopulation and geographic isolation, whereas older adults left behind face shortages of both financial and human resources to support their daily lives (Kim et al., 2022; Koo, 2021). Economically disadvantaged older adults are disproportionately concentrated in rural regions. Their vulnerability is compounded by lower income levels, limited educational attainment, poorer health, substandard housing conditions, fewer opportunities for social participation, restricted access to information technology, and limited availability of health care and welfare services (Kim & Kim, 2021; Lee & Lee, 2018). Consequently, older adults in rural communities face greater structural and social disadvantages than their urban counterparts, who generally benefit from better infrastructure and broader access to essential services (Cai & Lalani, 2022). These intersecting disadvantages underscore the urgency of rethinking how care is organized and delivered in rural Korea.

In response to these challenges, the Korean government introduced “Community-Based Integrated Care Plan (2018)” to address the widespread gaps in care services for older adults by strengthening community-based delivery (Ministry of Health and Welfare, 2018). More recently, this agenda incorporated a strong emphasis on information and communication technology (ICT)-based care services as a means of minimizing care gaps in an aging, information-oriented society (Shin et al., 2020). A range of digital tools has been adopted within home-based services, particularly for older adults living alone and receiving basic livelihood support. For example, emergency safety monitoring for older adults living alone on basic livelihood support installs gas and fire detectors, activity sensors, and emergency call buttons in homes to allow prompt responses to emergencies (Oh et al., 2022). Building on this, digital technology-integrated services such as AI/IoT-based care and emergency alarm systems for older adults living alone have been implemented as part of the Older Adults Customized Care Service, a nationwide social care program launched by the Korean government in 2020 to integrate and expand existing care initiatives for community-dwelling older adults (Lee & Huh, 2022; Seong & Song, 2023; Shin et al., 2020).

Parallel to these policy developments, the increasing adoption of smart care systems has spurred research into their effectiveness in improving care delivery. Studies examining users’ perceptions of digital technology report benefits such as enhanced information flow, increased work efficiency, improved care integration (Yutong et al., 2023), continuous monitoring, and support for independent living (Tian et al., 2024). However, empirical findings also highlight potential adverse effects, particularly the risk of exacerbating social isolation and loneliness, underscoring the importance of sustained human interaction to offset these risks (Tian et al., 2024). This concern is especially pronounced in rural areas, where geographic and infrastructural barriers limit access to respite care, transportation, and homemaker services (Ministry of Health and Welfare, 2025). In the Korean context, focus group discussions with rural older adults using smart home services identified “gradually adapting to smart home technology with the help of others” as a central theme (Baik et al., 2021). Although the literature underscores the need to integrate human interaction into smart care to balance efficiency with relational dimensions, it still raises a more fundamental question: what does “good care” mean in the context of digitally integrated environments in rural settings.

Research on good care tends to emphasize the importance of the relationship between care recipients and caregivers, as well as the values that emerge from this relational dynamic. Tronto’s ethical theory conceptualizes good care as a moral and political practice grounded in responsibility, interdependence, and empathy, rather than in abstract rules, principles, or moral judgment. This perspective is rooted in the premise that human beings are fundamentally relational and interdependent (Barnes et al., 2015; Tronto, 2013). From this standpoint, the quality of care is determined not merely by technical efficiency but by the extent to which care fosters relational understanding and solidarity between caregivers and recipients.

On this basis, the ethics of care delineates five interrelated phases, each involving specific values and responsibilities, and good care is achieved when these phases are enacted effectively and in relation to one another. “Caring about” involves attentive recognition and interpretation of needs. “Caring for” entails taking responsibility for acting and determining an appropriate response. “Care giving” refers to the competent and skillful provision of support, requiring sufficient resources and engagement. “Care receiving” concerns attunement to how recipients experience and evaluate the care provided. Finally, “caring with” encompasses the cultivation of trust, communication, respect, plurality, and solidarity in ways that sustain democratic forms of care (Barnes et al., 2015; Tronto, 2013). This ethical theory underscores the centrality of responsibility, social interconnectedness, and collaboration over abstract principles and moral judgment. However, under specific contextual conditions, particularly within digital technology-integrated care systems in rural communities, some studies suggest that technology tends to supplement this relational dimension (Chen et al., 2020; Gasteiger et al., 2021; Sharkey, 2014). Thus, it remains important to examine the extent to which human relationships continue to play a critical role even in depopulated regions where digital technology is introduced as a complementary means of care by exploring what constitutes good care.

Existing literature has shown the importance of the relational dimension in understanding what constitutes good care for older adults. Walsh and Shutes (2013) emphasized empathy, respect, and family-like relationships as critical to care quality, whereas From et al. (2009) highlighted the importance of being treated as a human being, respected as an individual, and supported by competent and committed caregivers. A systematic review reinforced these findings, identifying dignity, empowerment, social participation, and safety as recurring themes in older adults’ experiences of older adult care (Cleland et al., 2021). Collectively, this literature underscores that “good care” cannot be reduced to technical delivery but is deeply rooted in relational and processual aspects of caregiving. At the same time, the meaning of good care is not universal. Cultural variation is evident: Western frameworks tend to prioritize autonomy and independence, whereas Confucian traditions emphasize filial piety and the comprehensive fulfillment of older adults’ needs (Kyeong et al., 2025; Li et al., 2021). Moreover, perspectives differ depending on whether care is viewed from the perspectives of recipients or those of the providers and whether it is delivered in institutional or community-based settings. Studies that have considered providers’ perspectives point to the importance of family-like care, respect for individuality, and attention to both emotional and functional needs (Choi, 2010). These findings suggest that providers’ practices are not only instrumental but also deeply relational, shaping the extent to which older adults experience care as “good.” Despite these insights, little research has examined how service providers—those directly responsible for delivering and coordinating care—understand and operationalize good care within increasingly digitalized service systems. This gap is particularly critical in rural areas, where structural vulnerabilities such as social isolation and limited resources are compounded by the rapid introduction of smart care technologies. Existing studies have mainly focused on older adults’ perceptions of specific devices, offering only limited guidance for conceptualizing what constitutes quality care in digitally mediated contexts.

To address this gap, our study investigated how village care managers—frontline coordinators in rural communities who link older adults with health, welfare, and smart care resources—perceive and enact “good care” within the framework of smart integrated care. By focusing on their perspectives, this study examines how village care managers in depopulated rural areas of Korea conceptualize good care through their experiences managing and integrating health, welfare, and smart care services for older adults.

## Research design and methods

This study employed in-depth, semi-structured interviews to explore perceptions of good care among village care managers who coordinate health, welfare, and smart care services for older adults in rural Korea. As part of their role, these managers identified older residents in need through community meetings and home visits, connected them with local resources, and provided case management for older adults and their families across 15 depopulating rural districts in Jeongeup (Choi et al., 2025). A qualitative approach was particularly relevant given the rapid integration of digital technologies into care systems as a strategy to compensate for limited physical resources in population-declining areas. These areas, designated by Presidential Decree, are municipalities at risk of depopulation due to demographic changes and outmigration among both older adults and youth, with 89 municipalities officially classified as such in 2021 (Ministry of the Interior and Safety, 2021).

### Participants and data collection

In this study, purposive sampling was used to recruit participants. This non-probability sampling technique entails deliberately selecting participants whose characteristics are closely aligned with the research aims, on the premise that individuals with relevant experiences are likely to offer distinctive and meaningful perspectives (Campbell et al., 2020). Participants were 16 village care managers involved in the first-year pilot project of the “Smart Integrated Care City: From a Healthy City to a Care City, with a Focus on Jeongeup, Jeonbuk-do,” a research and development initiative by the Korea Health Industry Development Institute, which seeks to advance integrated care in rural settings.

The aim of this project was to implement integrated care for older adults by combining local health and welfare resources with joint case management undertaken in collaboration with village care managers. As part of the pilot, smart care technologies, particularly *Care-Net*, an integrated data platform designed to monitor, consolidate, and share information, were introduced to enhance efficiency, support service coordination, and expand accessibility in the population-decline area. In the Jeongeup pilot, rural adaptation strategies focused on tailoring services to environmental and infrastructural conditions while mobilizing local human resources to help residents identify and utilize local assets for sustainable care provision.

Two trained researchers conducted 16 in-person, individual in-depth interviews, each lasting 60 to 90 min. Interviews were semi-structured and utilized open-ended questions that allowed participants to respond freely. Interviews explored the everyday experiences of care managers in depopulated areas, their thoughts and needs related to caregiving, and their reflections on these experiences. Data analysis involved preparing field notes, transcripts, and other materials, followed by a thorough examination to draw conclusions. Interview questions covered demographic characteristics, caregiving service experience, daily routines, caregiving-related experiences, personal views on what constitutes good care, and experiences or observations of good care. The full interview guide is provided as Supplementary Material. Interviews were conducted between November 1 and December 30, 2023, and each session lasted approximately 60 to 90 min. All interviews were digitally audio-recorded with the consent of the participants. The recordings were transcribed verbatim by a professional transcriptionist and checked for accuracy by the researchers. This study was reviewed and approved by the institutional review board of Wonkwang University (WKUIRB-202311-SB-087).

### Analysis

The analysis procedure for this study adhered to the content analysis methodology proposed by Graneheim and Lundman (2004). First, transcripts and field notes were repeatedly reviewed to develop a comprehensive understanding of the phenomenon and to capture the overall essence of the data. Key thoughts and experiences were then extracted and condensed into meaning units. Tronto’s (1998) ethics of care was used as an analytic lens to understand how village care managers interpret and negotiate good care in technology-integrated care settings. Second, codes were systematically assigned, with careful consideration of the broader context. Third, an abstraction process was conducted in which differences and similarities among the identified codes were analyzed, leading to the classification of codes into subcategories and overarching categories. This process enabled the identification of latent content, including underlying meanings, which were subsequently developed into themes. For example, statements in which care managers described “serving older adults as if they were their own parents” were condensed into meaning units about emotional commitment, coded as “family-like empathy,” and later grouped under the overarching theme of empathetic care. Throughout the analysis, approximately one-quarter of the data was independently analyzed by each researcher to verify the consistency of the coding scheme. The remaining data were then analyzed, with the process being iteratively refined through multiple discussions until consensus was achieved. The overall data analysis was conducted using a qualitative content analysis approach, which involved the transcription of audio recordings followed by a systematic process of categorization and interpretation of the transcribed content.

For the analysis, the entire data set was thoroughly reviewed, and individual documents were read repeatedly to conduct the coding process. This resulted in the identification of 31 distinct concepts. Upon further examination and categorization, the 31 concepts were consolidated into five categories, which were then grouped into five overarching themes: empathetic care, respectful care, socially connected care, comprehensive professional care, and collaborative care.

## Results

Tables 1 and 2 present the demographic characteristics of the study participants (*n *= 16). The sample comprised six male participants (37.5%) and 10 female participants (62.5%). Regarding prior care work experience, nine participants (56.3%) reported prior experience in caregiving roles, whereas seven participants (43.7%) had no prior experience in the field. Participants ranged in age from 38 to 70 years, with a median age of 61.0 years. Participants’ levels of education spanned from a high school diploma to a Master’s degree, with a median educational level of a Bachelor’s degree. The duration of residency in the community varied significantly from 1.5 to 62 years, with a median of 31.0 years.

**Table 1:** Characteristics of village care managers who participated in the in-depth interviews.

Characteristics	*n* (%) or range (median response)
**Gender**	
Female	10 (63.0%)
Male	6 (38.0%)
**Age**	38–70 (61.0)
**Educational attainment**	High school—postgraduate school
**Previous care work experience**	
Yes	9 (56.3%)
No	7 (43.7%)
**Years of living in community**	1.5–62.0 (31.0)
**Total**	16

**Table 2:** Sociodemographic characteristics of village care managers by ID.

ID	Age	Gender	Educational attainment	Years of living in the community	Work experience in caregiving-related occupations
**1**	61	F	High school diploma	1.5	Care worker
**2**	67	F	2-year college	27	Care worker
**3**	55	M	4-year college	55	None
**4**	62	M	Master’s degree	30	None
**5**	60	F	High school diploma	8	None
**6**	62	F	2-year college	38	Disability support assistant
**7**	38	F	4-year college	5	Childcare teacher
**8**	68	M	4-year college	60	Director of a daycare center and childcare support center
**9**	54	F	4-year college	20	Care worker
**10**	70	M	4-year college	36	None
**11**	60	F	High school diploma	8	Care worker
**12**	44	F	Master’s degree	36	Care worker
**13**	61	F	4-year college	61	Care worker
**14**	57	M	4-year college	30	None
**15**	62	F	4-year college	62	None
**16**	62	M	Master’s degree	32	None

### Empathetic care

Good care is frequently characterized as being similar to or resembling the care provided by a family. According to one participant:

It’s about having a sincere heart to serve others as if they were your own parents. Quality care cannot be provided if you’re merely filling in for a few hours. Caregiving is not something that can be done mechanically or with strict precision; it requires genuine empathy and commitment. (Participant 14)

Participants in this study emphasized that quality care should be delivered with sincerity and genuine dedication, going beyond the basic responsibilities of duty. This heartfelt approach to caregiving, when considered collectively, aligns closely with the principles of family-like care. In sum, family-like care highlights the importance of sincerity and genuine dedication, particularly in addressing the emotional needs of those receiving care. As one participant emphasized, “Care is not just about tasks like cleaning or laundry. The key is to make them feel understood and comforted, so they genuinely feel cared for on an emotional level” (Participant 9). Caregiving services often focus on practical tasks such as housework, emergency alert systems, and instrumental aid. Participants consistently highlighted that good care must incorporate qualities such as empathy, dedication, reliability, understanding, intimacy, emotional bonds, and respect for individuality.

### Respectful care

In the caregiving process, respecting individuals’ traits and dignity is recognized as a crucial element. A common challenge is the tendency to treat older adults as a homogeneous group, overlooking their unique characteristics. This approach often leads to standardized care that lacks personalization and fails to address the specific needs of each person. To genuinely respect older adults, it is essential to view them as individuals and provide care tailored to their specific needs and preferences. As one participant emphasized, “The most effective caregiving is personalized, helping caregivers understand and meet the specific needs of older adults, such as accompanying them to appointments or offering companionship” (Participant 1). Older adults are often treated as a single homogeneous group; however, their circumstances and needs differ noticeably. The theme of respectful care captures participants’ emphasis on recognizing older adults as pluralistic and particular individuals, valuing their diverse contexts, experiences, and preferences rather than treating them as a homogenous group. For instance, participants noted that care needs often vary by gender; for instance, male older adults may require more in-home assistance due to less experience with household tasks and greater feelings of loneliness, given the higher number of women in older adults’ communities. Also, needs vary by health status: some may need more home care, whereas others may benefit more from social interactions to combat loneliness.

### Socially connected care

The significant decline in rural populations in Korea has led to a severe reduction in opportunities for social interaction, exacerbating loneliness among older adults. Participants consistently emphasized that social connection, facilitated through human engagement, which digital tools often fail to deliver, is essential for the well-being of older adults, particularly in rural areas where isolation is a pressing concern. One participant highlighted the depth of loneliness experienced by older adults, stating:

Older adults often spend their entire day watching cars, and people pass by. We brought them some cold water, and afterward, they began stopping by with a cucumber or squash, just to chat. It made me realize how much they missed human connection, spending their entire day simply watching people go by. (Participant 3)

This simple interaction revealed how deeply older adults crave human engagement and how even minimal social contact can make a significant difference in their lives. Another participant emphasized the importance of human engagement in preventing older adults from experiencing physical and emotional hardship, “I’ve noticed that when someone visits my mom, she becomes much more cheerful. Having someone to talk to helps her forget her pain and loneliness” (Participant 1).

This statement emphasizes the vital role of human engagement in reducing both physical and emotional distress among older adults. It highlights that social interaction fosters well-being, offering companionship and emotional resilience beyond what digital tools can provide.

Another critical reason why human engagement and social connection are essential is the heightened risk of severe isolation and unattended deaths among older adults living alone in rural areas. As one participant noted:

Almost all of them live alone. In our community, the houses are quite isolated from each other. If someone hasn’t been seen for a couple of days, it’s important to go and check on them to ensure they haven’t passed away or died alone without anyone knowing. Monitoring for such cases of isolation and potential unattended deaths seems crucial. (Participant 12)

This statement underscores the vital role of human engagement in preventing severe isolation and unattended deaths among older adults in rural areas. The physical separation of homes increases the risk of unnoticed emergencies, highlighting the limitations of formal care. A proactive, community-driven monitoring system is essential to ensure timely intervention and support.

### Comprehensive professional care

One community manager emphasized the growing need for skilled professionals in older-adult care as rural older adults face complex challenges, including aging-related issues, chronic illness, and service inaccessibility. Participants highlighted the necessity of a structured care system with trained administrators who possess specialized skills to navigate social services, manage bureaucratic complexities, and bridge digital gaps, ensuring more effective support. As one participant noted:

I have worked in administration for a long time. When residents face difficulties and struggle to navigate administrative procedures, I feel a sense of responsibility. Since I have extensive experience in this field, I believe I should study further and use my administrative knowledge to help them. (Participant 16)

This statement underscores the systemic barriers older adults face in navigating administrative processes, highlighting the potential need for professionalized support. Although community-based help is valuable, trained personnel with administrative expertise are essential to bridge accessibility gaps.

This need becomes even more critical with the integration of digital technologies into older care services, particularly in rural areas where many older adults live alone and often lack familiarity with digital devices. Without human involvement, the digital divide may further limit their access to essential services, underscoring the importance of a balanced approach that combines professional expertise with direct human support. As one community manager noted:

For these older individuals, the priority is not merely the benefits offered by the devices, but rather the community support and management required during the distribution and maintenance process (Participant 1). As another participant also noted, Helping older adults with technology upgrades has made me realize the importance of such support. When I put a participant’s phone on airplane mode for an interview, they asked me to turn it back on before leaving, as they wouldn’t know how to do it themselves. This moment highlighted the significant digital challenges they face. (Participant 3)

This excerpt underscores the need for professional support in bridging the digital divide among older adults, particularly in smart home systems. Their reliance on assistance with basic tasks highlights potential challenges in independent technology use. Although smart care technologies enhance accessibility, skilled guidance is essential for troubleshooting and sustainable adoption. To ensure good care, a comprehensive system must integrate both technical expertise and human-centered support.

### Collaborative care with local human resources

Interviews with participants revealed two key dimensions of collaborative care in addressing the complex and diverse welfare and health care needs of older adults in rural areas. The need for collaborative services arises from fragmented care systems, which lead to overlapping services and gaps in care delivery. One participant stated:

There is too much overlap in services. For example, when we visit older adults and ask, “Do you have any difficulties?” they often respond, “Oh, a caregiver comes, and someone from a center visits too.” They said, many of these services are nearly identical and redundant. (Participant 6)

Similarly, another participant emphasized the importance of a structured system to prevent gaps in care, stating, “A coordinated system is needed to prevent care gaps by linking community services rather than providing all services directly. A central manager plays a key role in ensuring seamless care, including emergency responses” (Participant 10). Participants highlighted overlapping services and care gaps, which stress the need for a coordinator to manage an integrated system. Good care requires interdisciplinary collaboration, combining digital technology and human interaction to connect welfare, health care, and medical services, especially in rural areas. One participant highlighted this by stating:

With mobile phones, wouldn’t it become easier for people in this community to participate in communal dining over the next 10 to 15 years? Most people live alone, so it would be helpful to have a digital-based service where they can check where meals are available and what’s on the menu. (Participant 13)

Although this example focuses on meal services for older adults, the response suggests that digital integration can improve access to service by enabling older adults to navigate community resources more efficiently. A higher level of service integration would increase the likelihood of meeting diverse needs, particularly when incorporated with digital solutions.

Secondly, the utilization of local human resources is crucial to supplement public care services, which frequently fall short of meeting the comprehensive needs of older adults as part of collaborative care. One participant emphasized the importance of collaborative efforts by local residents within the community, stating, “Care should be a shared responsibility within the community, not an individual task. Seeing care as both giving and receiving can strengthen mutual support among local residents” (Participant 15). This perspective highlights the limitations of a solely institutional approach to care and suggests that integrating local caregivers, medical professionals, and welfare providers can create a more responsive support network for older adults. Another participant also highlighted the necessity of locally driven solutions, stating, “While help from public institutions is necessary, it would be beneficial if smaller issues within the community could be resolved locally” (Participant 12). This response highlights the limitations of public care’s rigid structure and reach, emphasizing the vital role of community-based caregiving networks in providing flexible, context-specific support for older adults.

To improve utilization of human resources, another participant suggested peer caregiving, often referred to as “Older-adult Care by Older adults,” where older adults take on caregiving roles for their peers.

Who will care for those older adults? Younger people might try, but they often don’t fully understand the needs of older adults, which can make things awkward. They might also be less willing to take on the role. When familiar peers interact with older adults, there’s a better connection and more positive engagement. (Participant 6)

Notably, most of the care managers in this study were older adults themselves (median age = 61), and their own life experiences may have shaped their understanding of good care, enabling deeper empathy with the needs and emotions of their clients. This alignment between caregivers and recipients suggests that peer caregiving can be an effective approach, offering both practical support and emotional connection through shared experience, thereby bridging gaps in formal services and promoting older adults’ social and emotional well-being.

## Discussion and implications

This study explores how village care managers perceived quality care within the context of a smart care pilot program, analyzing their experiences and perspectives through the lens of Tronto’s ethics of care. Although Tronto’s framework centers on relational processes rather than technological infrastructures, it is used here to examine how digital tools become embedded in the relational work of care. Findings from our study indicate that village care managers regard multiple dimensions of good care within smart care systems as important, including empathetic and respectful care, social connected care, comprehensive professional care, and collaborative care with local human resources. These themes share a common insight that human engagement remains critical even in digitally integrated care environments. In particular, this finding reinforces that care is fundamentally relational and aligns with Tronto’s argument that caring and empathy arise from humans as interdependent beings (Barnes et al., 2015). More importantly, the themes suggest that participants’ accounts revealed an intuitive understanding of good care that aligns with the processes articulated in Tronto’s ethics of care. Their narratives reflected how the principles of attentiveness (caring about), responsibility (caring for), competence (caregiving), responsiveness (care receiving), and trust (caring with) guided their interpretations of what constitutes good care. Taken together, these reflections demonstrate that good care emerges when these processes are coherently enacted, emphasizing that the quality of care ultimately depends on how effectively such relational and ethical dimensions are embodied in practice.

The specific characteristics of each theme are discussed in detail below.

Empathetic care embodies intentional efforts to build relationships grounded in warmth, understanding, and genuine concern. This quality is particularly salient in the phase of “care giving,” which in Tronto’s ethics of care corresponds to competence. Consistent with previous studies, our findings indicate that good care extends beyond addressing physical or functional needs to encompass a sincere concern for each individual’s overall quality of life (Choi, 2010; Cleland et al., 2021; From et al., 2009; Seok et al., 2015). Participants described this attitude as “serving older adults as if they were their own parents,” which they regarded as essential to helping them feel understood and comforted. This perspective emphasizes that care should not be delivered mechanically but with emotional engagement and moral attentiveness. Empathy, as a core element of social care, enables practitioners to understand and share the emotions of care recipients, fostering communication and accurate assessment of needs that contribute to therapeutic change and improved social functioning (Moudatsou et al., 2020). Within Tronto’s framework, such practice represents competence when caregivers possess the time, resources, and skills required to provide adequate care. Moreover, an empathetic disposition is indispensable for realizing these conditions, as it allows caregivers to engage with older adults in ways that promote understanding and emotional support. Ultimately, this emotional resonance extends to the phase of responsiveness and culminates in caring-with, where mutual trust, solidarity, and collective responsibility are sustained (Barnes et al., 2015).

Respectful care emerged as another central dimension of good care in this study and is closely associated with the phase of “caring for” in the ethics of care, which entails taking responsibility for identifying specific needs and making appropriate decisions about how care should be provided. In this study, respectful care refers to recognizing each older adult as an individual worthy of dignity and respect. Tronto (2013) emphasized the importance of plurality and particularity, meaning that care must be responsive to the diverse and context-specific needs of care recipients. However, as Phelan (2008) observes, dominant social discourses often portray older adults as a homogeneous group defined by senility, cognitive decline, asexuality, and unemployability. Such representations reinforce age-based stereotypes and obscure the individuality and heterogeneity of older adults’ lived experiences. Against this backdrop, respectful care in this study involved acknowledging each older person’s distinct preferences, circumstances, and life histories rather than applying uniform routines or standardized procedures. Participants emphasized that understanding and responding to these differences enabled caregivers to provide more meaningful and person-centered support, thereby reinforcing the ethical principle that good care must begin with recognition of individual dignity and diversity.

Socially connected care and comprehensive professional care emerged as distinct dimensions of good care, representing themes uniquely identified in this study and rarely addressed in previous research. These themes were particularly evident in rural settings where digital technologies are increasingly integrated into care provision. They appeared to arise as participants reflected on the phase of caring for (responsibility), emphasizing the importance of recognizing and responding to older adults’ needs, particularly within the rural context examined in this study.

Compared to their urban counterparts, who benefit from greater access to infrastructure and services, older adults in rural communities are more vulnerable due to social isolation and limited resources. Structural barriers, including geographic isolation, inadequate transportation, sparse populations, and limited digital technology access, intensify these challenges for rural older adults (Cai & Lalani, 2022; Southerland et al., 2024; Theeke & Mallow, 2013). Within rural contexts, social isolation remains a persistent concern, not only in emotional terms such as loneliness and detachment but also in physical terms, as it can hinder timely responses during emergencies. Maintaining interpersonal connections is crucial for older adults’ overall well-being and safety (Barnes et al., 2015; Tronto, 2013). In this context, even simple human contact, such as casual conversations, can significantly enhance their emotional well-being and sense of connection. From this perspective, socially connected care is central to relationship-based care in rural communities. This suggests that technology alone cannot substitute for human involvement but needs to be integrated into practices that combine digital innovation with sustained human engagement, thereby ensuring that care remains both effective and morally responsive.

For the theme of comprehensive professional care for older adults in rural areas, participants mainly emphasized that a comprehensive approach integrating professionalized care and digital solutions is important. This aspect may be more closely related to the phase of competence (caregiving), which requires adequate skills, resources, and active participation to ensure effective care provision (Tronto, 1998). Our study suggests that, particularly in digitally integrated care settings, human personnel still need to actively manage and regulate digital services to ensure their effective implementation. Older adults often face significant barriers in accessing social services, as they struggle with bureaucratic complexities and the digital divide. Trained personnel are needed to guide them through service navigation, ensuring they receive appropriate care. This need is even more critical in smart home-based integrated care systems, where older adults may face difficulties operating devices or resolving technical issues. We identified cases where emergency alert services were available but underutilized due to a lack of familiarity. In Korea, many older adults have limited digital literacy due to low education levels and lifelong exposure to an analog environment (Kim et al., 2022). Struggles with digital tools often lead to negative emotional responses, such as anxiety and frustration (Brown & Markusson, 2019). As digital care resources expand, it is crucial to involve care workers with specialized skills, including digital proficiency and the ability to teach technology use. Simply offering new technologies, no matter how beneficial, may not be effective if older adults feel overwhelmed (Jo et al., 2021). Baik et al. (2021) found that older adults, especially those in rural areas, require time and external support to adapt to smart home services. Although initial adaptation is slow, with adequate assistance, they gradually become more comfortable with these technologies. These findings highlight the important role of human resources in supporting technological adaptation, reinforcing the importance of skilled caregiving personnel in digitally integrated care systems.

Lastly, our findings highlight the necessity of a collaborative care approach that integrates digital technology with local human resources to effectively address the complex welfare and health care needs of older adults in rural areas. This highlights the importance of solidarity, which centers on mutual support and trust as critical components of good care and corresponds to Tronto’s final phase of caring (Tronto, 1998). Despite ongoing efforts to enhance older adult care through digital solutions, previous studies have identified persistent service fragmentation as a major challenge in Korea, complicating the effectiveness of digital integration (Lim et al., 2020). This study’s findings align with prior research that has emphasized the negative impact of systemic fragmentation, including service duplication, gaps in care delivery, and difficulties in identifying and addressing unmet needs. Moreover, given the limitations of human and physical resources in elder care, participants underscored the importance of an interdisciplinary approach that combines digital solutions with local human resources to supplement public care services. This integration is particularly critical in smart home-based care models, where conventional public care services often fail to fully meet the diverse needs of older adults. By leveraging both technological advancements and community-based support, care systems can become more responsive, sustainable, and effective in addressing the unique challenges faced by aging populations in rural areas.

Our interview findings further highlight the central importance of human resources in rural elder care, particularly through peer-support models such as “older-adult care by older adults.” Most village care managers in this study were in a relatively higher age group, with a median age of 61, mirroring the demographic composition of the rural communities examined. However, their shared life experiences and cultural backgrounds not only shaped how they understood care but also made it easier for them to build rapport and maintain a sense of familiarity with care recipients. This generational proximity enabled more personalized and culturally attuned support, thereby strengthening the effectiveness of digitally integrated care in rural settings. Therefore, the strength of the participant sample indicates that the aging population can function as a valuable form of social capital in rural communities. Peer caregiving leverages these relational strengths to address service gaps and reinforce the social fabric of rural life. Taken together, the findings suggest that enhancing human connection through “older-adult care by older adults” offers a culturally resonant and sustainable strategy for rural elder care, transforming community relationships into a key source of caregiving capacity.

Overall, our findings on what constitutes good care, based on participants’ perspectives on smart home–based elder care in rural areas, revealed that Tronto’s ethics of care served as a conceptual lens through which participants articulated and interpreted their experiences. The identified themes reflected participants’ engagement with the moral qualities embedded in each phase of care, highlighting critical challenges unique to rural environments, such as loneliness, social isolation, diverse care needs, and fragmented service systems that often lead to duplication, omission, and care gaps. These findings suggest that the success of smart care initiatives depends not only on technological advancement but also on human-integrated care, where the expertise, interpersonal engagement, and local collaboration of village care managers are essential. Participants emphasized that “good care” in smart home–based systems must still embody empathetic relationships, respect, social connectedness, professionalism, and collaboration. Taken together, these dimensions underscore the need for a holistic and person-centered approach that seamlessly integrates digital innovation with human engagement to enhance responsiveness, coordination, and inclusivity in rural elder care. Therefore, rather than replacing community-based support, digital tools should enhance caregiving structures by fostering interdisciplinary cooperation among technology developers, health care providers, policymakers, and local communities. A relevant example is Germany’s *Digitale Dörfer* project, a rural digitalization initiative that provides online marketplaces, local news, delivery services, and communication platforms (Sept, 2020). Although digitalization addresses infrastructure limitations, the project highlights the indispensable role of human collaboration in designing responsive and community-specific care systems. This underscores the necessity of active engagement from local residents, policymakers, and care professionals to ensure that digital solutions complement rather than replace human caregiving. Thus, a sustainable rural elder care model must integrate institutional resources, informal caregiving networks, and technological innovations through continuous interdisciplinary collaboration. By fostering partnerships among government agencies, local communities, and digital service providers, such a model can create a comprehensive, adaptable, and responsive care system that effectively meets the evolving needs of older adults in rural areas.

The study has several limitations. First, there was difficulty in comparing the perceptions of older recipients and their families with those of care providers. Such a comparison would have offered a more comprehensive understanding of the diverse expectations and perceptions among these key stakeholders. Future research should address this gap by analyzing the views of all three groups to develop a more holistic understanding of care dynamics in rural areas. Also, this study examined participants’ opinions during the early phases of smart service integration, which may not fully capture how perceptions of quality care could evolve as the project advances from its initial stages to later phases. Previous research often relied on brief evaluation sessions using presentations or scenarios to explain or demonstrate the technology to participants, allowing them to share their opinions on technology-based care (Peek et al., 2017). Although this approach is useful for exploring potential models of care, it may not adequately reflect the lived experience of smart care, as participants’ views on the technology may shift over time. We recommend further exploration of the differences in perspectives between those who have had extended experience with technology and potential stakeholders who will be involved in the care system. Understanding these differences could provide critical insights for the future development of effective technologies.

## Conclusions

This study explores the perspectives of care providers on the increasing integration of digital-based care in rural Korea, a context characterized by rapid population aging, limited infrastructure, and persistent shortages in health care and care personnel. Care providers recognize digital technologies as a pragmatic solution to enhance the efficiency of service delivery and improve monitoring for older adults in resource-constrained environments. However, in discussions of “good care” grounded in Tronto’s ethics of care, village care managers underscore the limitations of technology in meeting the emotional and relational dimensions of care, reaffirming the enduring value of respectful, person-centered, and continuous care. They also stress the necessity of professional expertise to ensure the ethical and effective integration of digital tools. Although grounded in the specific context of rural Korea, the implications of this study extend to other countries facing similar demographic and structural challenges in rural areas. The findings suggest that in aging societies with sparse populations and underdeveloped care infrastructure, the development of care models that combine digital innovation with strong human engagement is not merely beneficial but essential. Therefore, rather than viewing technology and human-centered care as competing approaches, they should be understood as mutually reinforcing and jointly indispensable elements in constructing sustainable, contextually responsive care systems for older adults.

## Supplementary Material

igag018_Supplementary_Data

## Data Availability

The qualitative interview data supporting the findings of this study are not publicly available because they contain confidential information about participants and rural communities. De-identified transcripts or analytic materials cannot be shared to protect privacy and comply with institutional review board requirements. The study was not preregistered.
