# Supplementary material for: What constitutes good care in the context of depopulating rural Korea? Perspectives of village care managers amid digital transitions
Source: Innov Aging. 2026 Feb 15;10(4):igag018. doi: 10.1093/geroni/igag018 (PMC13049593; doi:10.1093/geroni/igag018)
Supplement: igag018_Supplementary_Data [file igag018_supplementary_data.zip › innage suppl Park, Jang, & Hwang.pdf]

Innovation in Aging **Supplementary Material: Park, Jang, & Hwang. (2026). What constitutes good care in the context of depopulating rural Korea?: Perspectives of village care managers amid digital transitions.**

Appendix: Interview Guide

Introduction

1. Please describe a typical day in your life.
2. What kinds of activities do you usually participate in, and what types of support or assistance do you feel you need in your daily life?
3. What motivated you to become a village care manager (pathway of entry)? Have you previously worked in any care-related services (work experience)?
4. What have been the most challenging and rewarding aspects of your work as a village care manager so far?

Main Questions

1. How would you define “care”? In the current context of smart care, what do you consider to be “good care”?
2. Do you think care is important in your community, particularly given that it is a region experiencing population decline?
3. In your view, who are the primary individuals and what are the key settings in the community where care is most needed?
4. What efforts do you make to build and maintain relationships with care recipients (older adults)?
5. Are you aware of any other care resources currently available in the community?

Closing

1. What do you think is necessary to provide care more effectively?
2. Finally, please feel free to share any additional thoughts or reflections you have about care.
